# Supplementary material for: Riparian vegetation composition and diversity shows resilience following cessation of livestock grazing in northeastern Oregon, USA
Source: PLoS One. 2022 Jan 21;17(1):e0250136. doi: 10.1371/journal.pone.0250136 (PMC8782521; doi:10.1371/journal.pone.0250136)
Supplement: S1 Fig — The ungrazed reaches on the left and the grazed reaches are on the right. (DOCX) [file pone.0250136.s001.docx]

**Supporting Information: S1 Fig.**

**Riparian vegetation composition and diversity shows resilience following cessation of livestock grazing in northeastern Oregon, USA.**

J Boone Kauffman^1*^, Greg Coleman^1^, Nick Otting^1^, Danna Lytjen^1^, Dana Nagy^1^ and Robert L. Beschta^2^

^1^Department of Fisheries, Wildlife and Conservation Sciences, Oregon State University Corvallis, Oregon, United States of America

^2^ Department of Forest Ecosystems and Society, Oregon State University, Corvallis, Oregon, United States of America 97331

**S1 Fig.** Paired photos of selected ungrazed (exclosed) and grazed reaches. The ungrazed reaches on the left and the grazed reaches are on the right.
