# Supplementary material for: Riparian vegetation composition and diversity shows resilience following cessation of livestock grazing in northeastern Oregon, USA
Source: PLoS One. 2022 Jan 21;17(1):e0250136. doi: 10.1371/journal.pone.0250136 (PMC8782521; doi:10.1371/journal.pone.0250136)
Supplement: S1 Table — (DOCX) [file pone.0250136.s002.docx]

**Supporting Information: S1 Table**

**Riparian vegetation composition and diversity shows resilience following cessation of livestock grazing in northeastern Oregon, USA.**

J Boone Kauffman^1*^, Greg Coleman^1^, Nick Otting^1^, Danna Lytjen^1^, Dana Nagy^1^ and Robert L. Beschta^2^

^1^Department of Fisheries, Wildlife and Conservation Sciences, Oregon State University Corvallis, Oregon, United States of America

^2^ Department of Forest Ecosystems and Society, Oregon State University, Corvallis, Oregon, United States of America 97331

**S1 Table**  Indicator categories, probability ranges, and indicator index values for species occurrence in wetlands (Wentworth et al. 1988).

| Wetland Indicator | Probability of Occurrence | Indicator |
| --- | --- | --- |
| Category | in Wetlands | Index |
| Obligate wetland (OBL) | >99% | 1 |
| Facultative wetland (FACW) | 67-99% | 2 |
| Facultative (FAC) | 34-66% | 3 |
| Facultative upland (FACU) | 1-33% | 4 |
| Upland (UPL) | <1% | 5 |
