# Supplementary material for: Riparian vegetation composition and diversity shows resilience following cessation of livestock grazing in northeastern Oregon, USA
Source: PLoS One. 2022 Jan 21;17(1):e0250136. doi: 10.1371/journal.pone.0250136 (PMC8782521; doi:10.1371/journal.pone.0250136)
Supplement: S3 Table — Numbers are the mean and standard error based upon their cover in at least 20 2 X 5 m plots for each study reach. (DOCX) [file pone.0250136.s004.docx]

**Supporting Information: S3 Table.**

**Riparian vegetation composition and diversity shows resilience following cessation of livestock grazing in northeastern Oregon, USA.**

J Boone Kauffman^1*^, Greg Coleman^1^, Nick Otting^1^, Danna Lytjen^1^, Dana Nagy^1^ and Robert L. Beschta^2^

^1^Department of Fisheries, Wildlife and Conservation Sciences, Oregon State University Corvallis, Oregon, United States of America

^2^ Department of Forest Ecosystems and Society, Oregon State University, Corvallis, Oregon, United States of America 97331

| **S3 Table.** Plant species cover (%) the 11 study reaches. Numbers are the mean and Standard error based upon their cover in at least 20 2 X 5 m plots for each study reach. | | | | | | | | | | |  |
| --- | --- | --- | --- | --- | --- | --- | --- | --- | --- | --- | --- |
|  | Bear | | | |  |  | Upper Swamp | | | | |
|  | Exclosed | | Grazed | |  |  | Exclosed | | Grazed | | |
|  | Mean | SE | Mean | SE |  |  | Mean | SE | Mean | SE | |
| Achillea millefolium L. | 2.0 | 0.8 | 4.0 | 1.3 |  | Achillea millefolium L. | 0.1 | 0.1 | 0.1 | 0.1 | |
| Agrostis stolonifera L. | 1.6 | 0.7 | 5.1 | 2.0 |  | Agrostis stolonifera L. | 17.9 | 2.6 | 8.0 | 2.0 | |
| Alopecurus aequalis Sobol. | 0.1 | 0.1 |  |  |  | Alopecurus aequalis Sobol. |  |  | 1.2 | 0.5 | |
| Alnus incana (L.) Moench ssp. tenuifolia (Nutt.) Breitung | 1.3 | 0.8 | 1.9 | 1.0 |  | Alnus incana (L.) Moench ssp. tenuifolia (Nutt.) Breitung | 12.9 | 3.6 | 6.6 | 2.5 | |
| Antennaria microphylla Rydb. | 0.0 | 0.0 |  |  |  | Carex geyeri Boott | 1.9 | 1.4 |  |  | |
| Aquilegia formosa Fisch. ex DC. | 0.0 | 0.0 | 0.1 | 0.1 |  | Carex pellita Michx. | 4.9 | 2.1 |  |  | |
| Arnica chamissonis Less. | 2.3 | 0.7 | 2.2 | 0.7 |  | Carex nebrascensis Dewey | 6.6 | 2.6 | 2.5 | 1.1 | |
| Symphyotrichum foliaceus Lindl. ex DC. | 5.5 | 1.5 | 2.5 | 0.7 |  | Carex utriculata Boott | 12.3 | 3.9 | 1.0 | 0.7 | |
| Bromus carinatus Hook. & Arn. | 2.7 | 1.0 |  |  |  | Cirsium remotifolium (Hook.) DC. var. odontolepis Petr. | 2.4 | 0.7 |  |  | |
| Carex pellita Michx. | 9.0 | 2.5 | 9.4 | 3.1 |  | Crataegus douglasii Lindl. | 0.4 | 0.4 |  |  | |
| Carex kelloggii W. Boott var. kelloggii | 2.2 | 1.0 | 0.2 | 0.2 |  | Dipsacus fullonum L. | 2.8 | 1.0 | 0.4 | 0.4 | |
| Carex microptera Mackenzie | 6.1 | 2.5 | 4.3 | 1.5 |  | Epilobium ciliatum Raf. | 3.4 | 0.9 | 0.9 | 0.4 | |
| Carex nebrascensis Dewey | 1.5 | 0.8 | 0.8 | 0.8 |  | Equisetum arvense L. | 2.1 | 1.1 | 1.0 | 0.7 | |
| Carex utriculata Boott | 8.6 | 2.7 | 0.9 | 0.7 |  | Erigeron philadelphicus L. | 15.4 | 2.8 | 0.3 | 0.2 | |
| Cirsium scariosum Nutt. | 0.0 | 0.0 | 0.1 | 0.1 |  | Fragaria virginiana Mill. ssp. platypetala (Rydb.) Staudt |  |  | 0.2 | 0.2 | |
| Deschampsia cespitosa (L.) Beauv. | 3.6 | 1.3 | 6.0 | 1.5 |  | Galium aparine L. | 3.1 | 1.7 | 1.1 | 0.7 | |
| Eleocharis palustris (L.) Roemer & J.A. Schultes | 0.1 | 0.1 | 0.6 | 0.5 |  | Galium bifolium S. Wats. | 0.2 | 0.2 |  |  | |
| Epilobium ciliatum Raf. | 0.6 | 0.3 | 0.1 | 0.1 |  | Geum macrophyllum Willd. | 0.9 | 0.4 | 0.1 | 0.1 | |
| Equisetum arvense L. | 9.4 | 2.7 | 18.7 | 3.2 |  | Glyceria striata (Lam.) A.S. Hitchc. | 9.2 | 2.1 | 0.1 | 0.1 | |
| Erigeron philadelphicus L. | 0.1 | 0.1 | 1.2 | 0.4 |  | Juncus balticus Willd. | 11.9 | 3.2 | 12.7 | 2.9 | |
| Festuca idahoensis Elmer | 0.4 | 0.3 |  |  |  | Juncus ensifolius Wikstr. | 1.1 | 0.5 |  |  | |
| Festuca rubra L. | 1.4 | 1.1 |  |  |  | Mentha canadensis L. | 2.6 | 0.8 | 0.1 | 0.1 | |
| Fragaria virginiana Mill. ssp. platypetala (Rydb.) Staudt | 9.8 | 2.6 | 5.7 | 1.8 |  | Erythranthe guttata (Fisch. ex DC.) G.L. Nesom | 0.8 | 0.3 |  |  | |
| Galium boreale L. | 2.0 | 1.1 | 0.9 | 0.5 |  | Myosotis scorpioides L. | 2.7 | 1.3 |  |  | |
| Glyceria striata (Lam.) A.S. Hitchc. | 0.1 | 0.1 | 0.1 | 0.1 |  | Phleum pratense L. | 3.9 | 1.0 | 1.0 | 0.5 | |
| Hypericum anagalloides Cham. & Schlecht. | 3.9 | 1.4 | 8.3 | 3.0 |  | Picea engelmannii Parry ex Engelm. |  |  | 2.6 | 1.7 | |
| Juncus balticus Willd. | 1.2 | 0.5 | 8.0 | 2.8 |  | Plantago major L. |  |  | 3.7 | 0.7 | |
| Juncus ensifolius Wikstr. | 0.4 | 0.3 | 0.5 | 0.3 |  | Potentilla gracilis Dougl. ex Hook. | 0.9 | 0.6 |  |  | |
| Juncus nevadensis S. Wats. | 0.0 | 0.0 | 0.2 | 0.2 |  | Poa pratensis L. | 4.1 | 1.3 | 9.3 | 2.8 | |
| Lolium multiflorum Lam. | 0.5 | 0.3 |  |  |  | Ribes cereum Dougl. |  |  | 0.1 | 0.1 | |
| Mentha canadensis L. | 0.3 | 0.2 | 0.5 | 0.3 |  | Ribes lacustre (Pers.) Poir. | 0.2 | 0.2 |  |  | |
| Mimulus moschatus Dougl. ex Lindl. | 0.1 | 0.1 |  |  |  | Rosa woodsii Lindl. |  |  | 0.4 | 0.4 | |
| Phleum pratense L. | 0.0 | 0.0 | 0.9 | 0.5 |  | Scirpus microcarpus J. Presl & C. Presl | 4.0 | 2.4 | 0.1 | 0.1 | |
| Pinus contorta Dougl. ex Loud. | 7.5 | 3.5 | 6.3 | 2.2 |  | Packera pseudaurea (Rydb.) W.A. Weber & Á. Löve | 0.2 | 0.2 | 0.4 | 0.3 | |
| Plantago major L. | 0.0 | 0.0 | 0.3 | 0.2 |  | Maianthemum stellatum (L.) Link | 0.1 | 0.1 |  |  | |
| Potentilla gracilis Dougl. ex Hook. | 0.2 | 0.2 | 0.3 | 0.3 |  | Eriocoma occidentalis (Thurb. ex S. Watson) Romasch. | 0.1 | 0.1 |  |  | |
| Poa pratensis L. | 10.0 | 2.1 | 15.1 | 3.2 |  | Symphoricarpos albus (L.) Blake | 0.3 | 0.3 |  |  | |
| Prunella vulgaris L. | 2.7 | 1.5 | 7.7 | 2.3 |  | Taraxacum officinale G.H. Weber ex Wiggers |  |  | 0.9 | 0.4 | |
| Ranunculus macounii Britt. | 0.2 | 0.2 | 3.8 | 1.9 |  | Trifolium longipes Nutt. | 1.8 | 1.0 | 3.2 | 1.4 | |
| Rumex acetosella L. |  |  | 0.2 | 0.2 |  | Trifolium repens L. | 0.7 | 0.4 | 8.7 | 2.1 | |
| Salix geyeriana Anderss. |  |  | 0.8 | 0.4 |  | Veronica americana Schwein. ex Benth. |  |  | 3.4 | 1.7 | |
| Salix lasiandra Benth. var. caudata (Nutt.) Sudw. | 0.1 | 0.1 | 0.3 | 0.3 |  | Veronica anagallis-aquatica L. | 0.1 | 0.1 | 0.9 | 0.4 | |
| Scirpus microcarpus J. Presl & C. Presl | 1.1 | 1.1 | 1.0 | 0.8 |  | Veratrum californicum Dur. | 0.8 | 0.4 | 0.3 | 0.2 | |
| Packera pseudaurea (Rydb.) W.A. Weber & Á. Löve | 0.9 | 0.6 |  |  |  |  |  |  |  |  | |
| Solidago lepida DC. | 0.7 | 0.5 |  |  |  |  |  |  |  |  | |
| Sidalcea oregana (Nutt. ex Torr. & Gray) Gray |  |  | 0.2 | 0.2 |  |  |  |  |  |  | |
| Stellaria longipes Goldie | 0.4 | 0.2 |  |  |  |  |  |  |  |  | |
| Eriocoma occidentalis (Thurb. ex S. Watson) Romasch. | 0.2 | 0.2 | 0.5 | 0.5 |  |  |  |  |  |  | |
| Taraxacum officinale G.H. Weber ex Wiggers | 0.4 | 0.3 | 4.4 | 1.6 |  |  |  |  |  |  | |
| Trifolium longipes Nutt. | 9.3 | 2.4 | 8.5 | 2.4 |  |  |  |  |  |  | |
| Trifolium repens L. | 1.1 | 0.8 | 16.2 | 3.7 |  |  |  |  |  |  | |

|  | Camas | | | |  |  | Camp Creek | | | |
| --- | --- | --- | --- | --- | --- | --- | --- | --- | --- | --- |
|  | Exclosed | | Grazed | |  |  | Exclosed | | Grazed | |
|  | Mean | SE | Mean | SE |  |  | Mean | SE | Mean | SE |
| Achillea millefolium L. | 2.5 | 1.1 | 3.4 | 1.0 |  | Aconitum columbianum Nutt. |  |  | 0.4 | 0.3 |
| Agrostis stolonifera L. | 5.0 | 2.0 | 4.8 | 1.0 |  | Achillea millefolium L. | 1.0 | 0.5 | 1.7 | 0.7 |
| Alopecurus aequalis Sobol. | 0.1 | 0.1 |  |  |  | Agrostis stolonifera L. | 3.3 | 0.8 | 5.6 | 2.1 |
| Alopecurus pratensis L. |  |  | 2.7 | 1.8 |  | Alopecurus aequalis Sobol. |  |  | 0.0 | 0.0 |
| Arnica chamissonis Less. | 0.4 | 0.2 | 0.3 | 0.2 |  | Alnus incana (L.) Moench ssp. tenuifolia (Nutt.) Breitung | 30.5 | 4.4 | 10.1 | 3.8 |
| Symphyotrichum foliaceus Lindl. ex DC. | 0.7 | 0.3 | 0.2 | 0.2 |  | Angelica arguta Nutt. | 6.3 | 2.5 | 0.1 | 0.1 |
| Carex geyeri Boott |  |  | 0.5 | 0.5 |  | Aquilegia formosa Fisch. ex DC. | 0.1 | 0.1 | 0.4 | 0.4 |
| Carex pellita Michx. | 7.3 | 3.0 | 5.6 | 2.0 |  | Aster foliaceus Lindl. ex DC. | 2.6 | 0.6 | 9.4 | 1.8 |
| Carex kelloggii W. Boott var. kelloggii | 3.9 | 1.8 | 0.6 | 0.4 |  | Carex pellita Michx. | 0.2 | 0.2 | 0.2 | 0.2 |
| Carex microptera Mackenzie | 0.1 | 0.1 |  |  |  | Carex kelloggii W. Boott var. kelloggii | 3.9 | 1.0 | 2.8 | 0.8 |
| Carex utriculata Boott | 2.4 | 2.4 |  |  |  | Carex microptera Mackenzie | 4.4 | 0.9 | 0.3 | 0.2 |
| Cirsium remotifolium (Hook.) DC. var. odontolepis Petr. | 0.4 | 0.3 |  |  |  | Carex praegracilis W. Boott | 0.3 | 0.2 | 0.3 | 0.3 |
| Deschampsia cespitosa (L.) Beauv. | 2.3 | 1.6 | 0.1 | 0.1 |  | Cirsium scariosum Nutt. | 0.1 | 0.1 |  |  |
| Eleocharis palustris (L.) Roemer & J.A. Schultes | 2.1 | 1.2 | 4.0 | 1.9 |  | Dactylis glomerata L. |  |  | 0.1 | 0.1 |
| Epilobium ciliatum Raf. | 3.7 | 0.8 | 1.9 | 0.5 |  | Eleocharis palustris (L.) Roemer & J.A. Schultes | 1.7 | 0.9 |  |  |
| Equisetum arvense L. | 3.9 | 1.7 | 1.7 | 0.6 |  | Epilobium ciliatum Raf. | 0.4 | 0.2 | 1.7 | 0.5 |
| Equisetum laevigatum A. Braun | 1.0 | 0.6 |  |  |  | Equisetum arvense L. | 9.4 | 2.9 | 6.0 | 1.7 |
| Erigeron philadelphicus L. | 4.5 | 1.8 | 0.7 | 0.4 |  | Equisetum laevigatum A. Braun | 0.1 | 0.1 |  |  |
| Festuca rubra L. | 3.9 | 1.9 |  |  |  | Erigeron philadelphicus L. | 10.9 | 2.2 | 0.7 | 0.3 |
| Fragaria virginiana Mill. ssp. platypetala (Rydb.) Staudt | 0.1 | 0.1 | 0.4 | 0.3 |  | Fragaria virginiana Mill. ssp. platypetala (Rydb.) Staudt | 7.9 | 2.8 | 10.7 | 3.5 |
| Galium boreale L. | 2.1 | 0.7 | 0.6 | 0.3 |  | Galium aparine L. | 14.9 | 3.0 | 2.2 | 1.1 |
| Glyceria striata (Lam.) A.S. Hitchc. | 0.7 | 0.3 | 0.5 | 0.2 |  | Galium boreale L. | 0.9 | 0.5 | 0.3 | 0.2 |
| Juncus balticus Willd. | 5.0 | 2.2 | 3.2 | 1.5 |  | Glyceria striata (Lam.) A.S. Hitchc. | 4.2 | 0.9 | 5.1 | 1.2 |
| Juncus ensifolius Wikstr. | 0.6 | 0.3 | 0.3 | 0.3 |  | Heracleum maximum W. Bartram | 7.4 | 2.2 |  |  |
| Lolium multiflorum Lam. | 0.5 | 0.2 | 0.3 | 0.2 |  | Hypericum anagalloides Cham. & Schlecht. | 0.1 | 0.1 |  |  |
| Acmispon americanus (Nutt.) Rydb. |  |  | 4.0 | 2.6 |  | Juncus balticus Willd. | 24.4 | 4.6 | 15.7 | 3.6 |
| Lupinus leucophyllus Dougl. ex Lindl. | 1.9 | 1.2 | 1.3 | 0.7 |  | Juncus ensifolius Wikstr. | 1.1 | 0.5 | 0.5 | 0.3 |
| Mentha canadensis L. | 4.9 | 1.1 | 5.1 | 1.4 |  | Juncus nevadensis S. Wats. | 0.5 | 0.3 |  |  |
| Medicago lupulina L. |  |  | 0.3 | 0.2 |  | Juniperus occidentalis Hook. | 0.4 | 0.4 |  |  |
| Erythranthe guttata (Fisch. ex DC.) G.L. Nesom | 1.5 | 0.7 | 0.6 | 0.4 |  | Larix occidentalis Nutt. | 2.8 | 2.0 | 4.5 | 2.5 |
| Phleum pratense L. | 3.8 | 1.2 | 6.8 | 2.1 |  | Lolium multiflorum Lam. | 0.9 | 0.6 |  |  |
| Pinus contorta Dougl. ex Loud. | 1.0 | 1.0 | 1.9 | 1.3 |  | Acmispon americanus (Nutt.) Rydb. | 0.2 | 0.2 |  |  |
| Plantago lanceolata L. |  |  | 0.3 | 0.2 |  | Mentha canadensis L. | 6.7 | 1.9 | 3.3 | 1.2 |
| Plantago major L. |  |  | 0.8 | 0.4 |  | Erythranthe guttata (Fisch. ex DC.) G.L. Nesom | 0.5 | 0.3 | 0.5 | 0.3 |
| Potentilla gracilis Dougl. ex Hook. | 1.8 | 0.6 | 3.1 | 0.8 |  | Mimulus moschatus Dougl. ex Lindl. | 0.1 | 0.1 | 0.4 | 0.2 |
| Poa pratensis L. | 19.4 | 4.2 | 15.1 | 3.3 |  | Phleum pratense L. | 7.6 | 1.3 | 5.3 | 0.9 |
| Prunella vulgaris L. | 0.1 | 0.1 | 0.4 | 0.3 |  | Pinus contorta Dougl. ex Loud. | 5.8 | 2.7 | 2.0 | 2.0 |
| Rumex acetosella L. |  |  | 0.1 | 0.1 |  | Platanthera dilatata (Pursh) Lindl. ex Beck | 0.2 | 0.2 |  |  |
| Rumex crispus L. | 0.6 | 0.4 | 0.1 | 0.1 |  | Plantago major L. |  |  | 0.7 | 0.3 |
| Salix boothii Dorn | 2.6 | 2.1 |  |  |  | Potentilla gracilis Dougl. ex Hook. | 0.2 | 0.2 | 0.4 | 0.4 |
| Salix geyeriana Anderss. | 0.1 | 0.1 | 0.5 | 0.4 |  | Polemonium occidentale Greene | 0.4 | 0.2 |  |  |
| Salix lasiandra Benth. var. caudata (Nutt.) Sudw. | 0.1 | 0.1 | 1.9 | 1.5 |  | Poa pratensis L. | 33.5 | 3.1 | 50.8 | 3.1 |
| Scirpus microcarpus J. Presl & C. Presl | 4.6 | 1.9 | 6.7 | 2.4 |  | Prunella vulgaris L. | 0.7 | 0.3 | 7.0 | 1.2 |
| Packera pseudaurea (Rydb.) W.A. Weber & Á. Löve |  |  | 0.7 | 0.5 |  | Ribes cereum Dougl. | 0.3 | 0.2 | 0.7 | 0.5 |
| Sidalcea oregana (Nutt. ex Torr. & Gray) Gray | 0.4 | 0.3 | 0.3 | 0.2 |  | Ribes hudsonianum Richards. | 2.6 | 1.3 | 1.9 | 0.9 |
| Maianthemum stellatum (L.) Link | 0.1 | 0.1 |  |  |  | Salix lasiandra Benth. var. caudata (Nutt.) Sudw. | 0.3 | 0.3 |  |  |
| Solidago lepida DC. | 0.7 | 0.4 |  |  |  | Scirpus microcarpus J. Presl & C. Presl | 1.0 | 0.8 | 4.2 | 1.7 |
| Eriocoma occidentalis (Thurb. ex S. Watson) Romasch. |  |  | 0.1 | 0.1 |  | Packera pseudaurea (Rydb.) W.A. Weber & Á. Löve | 3.9 | 0.9 | 2.6 | 1.0 |
| Taraxacum officinale G.H. Weber ex Wiggers | 0.2 | 0.2 | 0.2 | 0.2 |  | Sidalcea oregana (Nutt. ex Torr. & Gray) Gray | 0.1 | 0.1 |  |  |
| Thermopsis montana Nutt. | 2.2 | 1.4 |  |  |  | Maianthemum stellatum (L.) Link | 4.8 | 2.4 | 0.4 | 0.3 |
| Thalictrum occidentale Gray | 0.5 | 0.3 | 0.1 | 0.1 |  | Symphoricarpos albus (L.) Blake |  |  | 5.9 | 2.8 |
| Trifolium cyathiferum Lindl. | 0.5 | 0.3 | 0.3 | 0.2 |  | Taraxacum officinale G.H. Weber ex Wiggers | 1.4 | 0.4 | 3.8 | 1.1 |
| Thermopsis montana Nutt. |  |  | 2.7 | 1.8 |  | Trifolium longipes Nutt. | 21.2 | 4.3 | 25.7 | 3.9 |
| Trifolium repens L. | 0.9 | 0.5 | 7.0 | 2.1 |  | Trifolium repens L. |  |  | 2.4 | 1.2 |
| Veratrum californicum Dur. | 0.1 | 0.1 | 0.4 | 0.4 |  | Urtica dioica L. | 0.1 | 0.1 |  |  |
|  |  |  |  |  |  | Viola adunca Sm. | 0.5 | 0.3 |  |  |
|  |  |  |  |  |  | Vicia americana Muhl. ex Willd. | 0.5 | 0.2 |  |  |

|  | Chesnimnus | | | |  |  | Tex Creek | | | | |
| --- | --- | --- | --- | --- | --- | --- | --- | --- | --- | --- | --- |
|  | Exclosed | | Grazed | |  |  | Exclosed | | | Grazed | |
|  | Mean | SE | Mean | SE |  |  | Mean | SE | Mean | | SE |
| Aconitum columbianum Nutt. |  |  | 0.1 | 0.1 |  | Aconitum columbianum Nutt. | 0.1 | 0.1 |  | |  |
| Acer glabrum Torr. |  |  | 1.1 | 1.1 |  | Achillea millefolium L. | 0.4 | 0.4 | 1.0 | | 0.5 |
| Achillea millefolium L. |  |  | 0.3 | 0.2 |  | Agrostis stolonifera L. | 4.3 | 1.5 | 4.4 | | 1.6 |
| Agrostis stolonifera L. | 0.3 | 0.2 | 0.4 | 0.2 |  | Agastache urticifolia (Benth.) Kuntze |  |  | 0.3 | | 0.3 |
| Alopecurus aequalis Sobol. | 0.3 | 0.3 | 0.3 | 0.2 |  | Alnus incana (L.) Moench ssp. tenuifolia (Nutt.) Breitung | 53.8 | 6.1 | 40.4 | | 7.2 |
| Alnus incana (L.) Moench ssp. tenuifolia (Nutt.) Breitung | 1.3 | 1.0 | 5.2 | 3.3 |  | Amelanchier alnifolia (Nutt.) Nutt. ex M. Roemer |  |  | 0.3 | | 0.3 |
| Alopecurus pratensis L. |  |  | 0.5 | 0.3 |  | Angelica arguta Nutt. | 0.3 | 0.2 |  | |  |
| Angelica arguta Nutt. | 0.9 | 0.9 | 2.9 | 1.1 |  | Antennaria microphylla Rydb. | 0.8 | 0.8 |  | |  |
| Arnica cordifolia Hook. | 2.3 | 1.6 | 1.2 | 0.5 |  | Symphyotrichum foliaceus Lindl. ex DC. | 11.4 | 4.1 | 2.1 | | 1.0 |
| Artemisia ludoviciana Nutt. | 0.8 | 0.4 |  |  |  | Cardamine cordifolia Gray | 0.3 | 0.3 |  | |  |
| Carex pellita Muhl. ex Willd. pellita. | 4.6 | 2.7 |  |  |  | Carex geyeri Boott | 2.5 | 2.5 |  | |  |
| Carex microptera Mackenzie | 0.3 | 0.2 | 0.6 | 0.4 |  | Carex pellita Muhl. ex Willd. | 0.9 | 0.9 |  | |  |
| Carex utriculata Boott | 0.9 | 0.9 |  |  |  | Carex microptera Mackenzie | 2.2 | 1.0 | 3.8 | | 2.1 |
| Cirsium remotifolium (Hook.) DC. var. odontolepis Petr. | 0.2 | 0.2 |  |  |  | Carex praegracilis W. Boott |  |  | 0.0 | | 0.0 |
| Cornus sericea L. |  |  | 1.8 | 1.8 |  | Cornus sericea L. | 2.4 | 0.9 | 1.5 | | 1.2 |
| Crataegus douglasii Lindl. | 5.5 | 2.9 | 7.6 | 3.5 |  | Crataegus douglasii Lindl. | 0.6 | 0.6 |  | |  |
| Deschampsia cespitosa (L.) Beauv. | 0.2 | 0.2 | 0.1 | 0.1 |  | Epilobium ciliatum Raf. | 0.9 | 0.6 | 0.3 | | 0.3 |
| Eleocharis palustris (L.) Roemer & J.A. Schultes | 1.1 | 0.6 | 0.5 | 0.3 |  | Equisetum arvense L. | 9.4 | 3.4 | 8.3 | | 3.7 |
| Epilobium ciliatum Raf. | 0.5 | 0.3 | 0.2 | 0.2 |  | Festuca arundinacea Schreb. | 0.3 | 0.3 | 0.3 | | 0.2 |
| Equisetum arvense L. | 30.0 | 4.1 | 0.6 | 0.4 |  | Galium aparine L. | 7.6 | 2.8 | 0.8 | | 0.4 |
| Erigeron philadelphicus L. | 4.7 | 1.0 |  |  |  | Galium boreale L. |  |  | 0.9 | | 0.5 |
| Fragaria virginiana Mill. ssp. platypetala (Rydb.) Staudt | 0.4 | 0.3 | 1.7 | 0.9 |  | Geum macrophyllum Willd. | 0.3 | 0.2 | 0.2 | | 0.2 |
| Galium aparine L. | 1.9 | 1.5 |  |  |  | Geranium richardsonii Fisch. & Trautv. |  |  | 0.3 | | 0.2 |
| Glyceria striata (Lam.) A.S. Hitchc. | 1.1 | 0.6 | 0.2 | 0.2 |  | Glyceria striata (Lam.) A.S. Hitchc. | 9.5 | 2.2 | 2.9 | | 1.6 |
| Juncus balticus Willd. |  |  | 0.1 | 0.1 |  | Heracleum maximum W. Bartram | 7.7 | 2.5 |  | |  |
| Juncus ensifolius Wikstr. | 0.3 | 0.2 | 0.1 | 0.1 |  | Juncus balticus Willd. |  |  | 3.0 | | 2.2 |
| Lolium multiflorum Lam. | 4.4 | 2.6 |  |  |  | Juncus nevadensis S. Wats. |  |  | 0.4 | | 0.2 |
| Mentha canadensis L.  . | 1.8 | 0.5 | 0.8 | 0.3 |  | Juniperus occidentalis Hook. |  |  | 0.3 | | 0.3 |
| Monardella odoratissima Benth. | 0.2 | 0.2 |  |  |  | Ligusticum grayi Coult. & Rose |  |  | 0.0 | | 0.0 |
| Myosotis scorpioides L. | 7.1 | 2.2 | 8.4 | 2.3 |  | Mentha canadensis L. | 6.5 | 1.9 | 3.1 | | 1.2 |
| Phleum pratense L. | 3.3 | 1.0 | 1.7 | 0.5 |  | Medicago lupulina L. |  |  | 4.3 | | 2.1 |
| Pinus contorta Dougl. ex Loud. | 0.2 | 0.2 |  |  |  | Myosotis scorpioides L. | 2.1 | 2.1 |  | |  |
| Plantago major L. | 0.7 | 0.3 | 4.7 | 1.0 |  | Osmorhiza occidentalis (Nutt. ex Torr. & Gray) Torr. |  |  | 0.4 | | 0.4 |
| Populus trichocarpa Torr. & A. Gray | 4.9 | 2.6 |  |  |  | Phleum pratense L. | 0.1 | 0.1 | 0.4 | | 0.2 |
| Potentilla gracilis Dougl. ex Hook. | 0.8 | 0.4 | 0.5 | 0.3 |  | Pinus ponderosa P.& C. Lawson | 2.8 | 1.8 | 5.0 | | 3.5 |
| Poa pratensis L. | 21.4 | 4.3 | 27.2 | 4.1 |  | Potentilla gracilis Dougl. ex Hook. | 0.3 | 0.2 |  | |  |
| Pseudotsuga menziesii (Mirbel) Franco |  |  | 11.0 | 3.2 |  | Poa pratensis L. | 16.1 | 3.6 | 14.3 | | 3.1 |
| Ribes aureum Pursh |  |  | 1.1 | 0.8 |  | Pseudotsuga menziesii (Mirbel) Franco | 2.1 | 2.1 |  | |  |
| Ribes cereum Dougl. |  |  | 2.8 | 1.7 |  | Ribes hudsonianum Richards. | 9.8 | 3.2 | 6.5 | | 2.5 |
| Ribes hudsonianum Richards. | 0.2 | 0.2 | 2.5 | 1.6 |  | Ribes lacustre (Pers.) Poir. |  |  | 3.0 | | 2.0 |
| Ribes lacustre (Pers.) Poir. |  |  | 0.4 | 0.3 |  | Rosa woodsii Lindl. |  |  | 0.9 | | 0.8 |
| Rosa woodsii Lindl. | 1.3 | 1.3 | 0.7 | 0.7 |  | Rumex crispus L. | 0.9 | 0.6 |  | |  |
| Rumex crispus L. | 0.1 | 0.1 |  |  |  | Salix bebbiana Sarg. | 0.1 | 0.1 |  | |  |
| Salix × fragilis L. | 17.7 | 4.9 |  |  |  | Salix geyeriana Anderss. |  |  | 0.1 | | 0.1 |
| Salix bebbiana Sarg. | 0.6 | 0.6 |  |  |  | Salix lasiandra Benth. var. caudata (Nutt.) Sudw. | 0.3 | 0.3 | 0.3 | | 0.2 |
| Salix exigua Nutt. var. exigua | 3.1 | 2.3 |  |  |  | Maianthemum stellatum (L.) Link | 4.4 | 2.6 | 2.1 | | 1.1 |
| Salix lasiandra Benth. var. caudata (Nutt.) Sudw. | 2.5 | 1.8 | 0.2 | 0.2 |  | Eriocoma occidentalis (Thurb. ex S. Watson) Romasch. |  |  | 0.1 | | 0.1 |
| Scirpus microcarpus J. Presl & C. Presl | 1.8 | 1.5 |  |  |  | Symphoricarpos albus (L.) Blake | 4.6 | 2.0 | 7.6 | | 2.4 |
| Sidalcea oregana (Nutt. ex Torr. & Gray) Gray | 0.1 | 0.1 |  |  |  | Taraxacum officinale G.H. Weber ex Wiggers | 0.2 | 0.2 | 0.7 | | 0.3 |
| Solidago lepida DC. | 0.1 | 0.1 |  |  |  | Thalictrum occidentale Gray |  |  | 0.4 | | 0.2 |
| Eriocoma occidentalis (Thurb. ex S. Watson) Romasch. | 0.5 | 0.3 |  |  |  | Trifolium longipes Nutt. |  |  | 1.3 | | 0.7 |
| Symphoricarpos albus (L.) Blake | 1.2 | 0.8 | 0.6 | 0.4 |  | Trifolium repens L. |  |  | 0.6 | | 0.4 |
| Trifolium repens L. | 1.8 | 0.9 | 10.9 | 3.3 |  | Veronica americana Schwein. ex Benth. | 1.2 | 0.7 |  | |  |
| Veronica americana Schwein. ex Benth. | 0.7 | 0.5 |  |  |  | Vicia americana Muhl. ex Willd. | 1.9 | 1.9 |  | |  |
| Veratrum californicum Dur. | 0.4 | 0.3 | 0.1 | 0.1 |  |  |  |  |  | |  |

|  | Devil's | | | |  |  | Summit Creek | | | |
| --- | --- | --- | --- | --- | --- | --- | --- | --- | --- | --- |
|  | Exclosed | | Grazed | |  |  | Exclosed | | Grazed | |
|  | Mean | SE | Mean | SE |  |  | Mean | SE | Mean | SE |
| Achillea millefolium L. | 1.5 | 0.6 | 1.3 | 0.5 |  | Achillea millefolium L. | 1.6 | 0.7 | 2.0 | 0.9 |
| Agrostis stolonifera L. | 15.0 | 2.7 | 1.8 | 0.5 |  | Agrostis stolonifera L. |  |  | 0.3 | 0.3 |
| Alopecurus aequalis Sobol. | 0.4 | 0.4 | 0.3 | 0.3 |  | Alnus incana (L.) Moench ssp. tenuifolia (Nutt.) Breitung | 13.6 | 4.2 | 0.1 | 0.1 |
| Alnus incana (L.) Moench ssp. tenuifolia (Nutt.) Breitung | 9.7 | 2.7 | 0.5 | 0.5 |  | Antennaria microphylla Rydb. | 1.0 | 0.7 | 0.4 | 0.3 |
| Angelica arguta Nutt. | 0.8 | 0.4 | 0.1 | 0.1 |  | Arnica chamissonis Less. | 2.6 | 1.1 |  |  |
| Carex geyeri Boott | 3.4 | 1.4 |  |  |  | Artemisia ludoviciana Nutt. | 0.1 | 0.1 |  |  |
| Carex pellita Michx. | 6.4 | 2.5 |  |  |  | Artemisia tridentata Nutt. ssp. vaseyana (Rydb.) Beetle |  |  | 3.8 | 2.0 |
| Carex kelloggii W. Boott var. kelloggii | 1.3 | 0.5 |  |  |  | Symphyotrichum foliaceus Lindl. ex DC. | 0.2 | 0.2 | 1.5 | 0.8 |
| Carex microptera Mackenzie | 1.6 | 0.6 | 0.2 | 0.2 |  | Carex pellita Muhl. ex Willd.pellita. | 16.1 | 3.9 | 8.5 | 3.3 |
| Carex utriculata Boott | 0.3 | 0.3 |  |  |  | Carex microptera Mackenzie | 0.9 | 0.6 | 0.8 | 0.8 |
| Cirsium remotifolium (Hook.) DC. var. odontolepis Petr. | 0.2 | 0.2 |  |  |  | Carex nebrascensis Dewey | 18.6 | 4.5 | 5.4 | 1.7 |
| Cornus sericea L. | 0.2 | 0.2 |  |  |  | Carex utriculata Boott | 13.9 | 3.8 |  |  |
| Deschampsia cespitosa (L.) Beauv. | 0.2 | 0.2 |  |  |  | Ericameria nauseosa (Pall. ex Pursh) G.L. Nesom & G.I. Baird |  |  | 0.3 | 0.3 |
| Eleocharis palustris (L.) Roemer & J.A. Schultes | 0.6 | 0.6 |  |  |  | Cirsium callilepis (Greene) Jepson | 0.5 | 0.4 |  |  |
| Epilobium ciliatum Raf. | 2.7 | 1.3 | 1.0 | 0.4 |  | Eleocharis palustris (L.) Roemer & J.A. Schultes | 7.7 | 2.5 | 3.7 | 1.0 |
| Equisetum arvense L. | 4.7 | 2.1 | 3.4 | 1.4 |  | Epilobium ciliatum Raf. | 2.5 | 1.4 | 0.3 | 0.2 |
| Fragaria virginiana Mill. ssp. platypetala (Rydb.) Staudt | 1.7 | 0.9 | 1.7 | 0.9 |  | Equisetum arvense L. |  |  | 0.2 | 0.2 |
| Galium aparine L. | 1.9 | 0.9 |  |  |  | Erigeron philadelphicus L. | 0.7 | 0.5 | 0.2 | 0.2 |
| Galium boreale L. |  |  | 0.1 | 0.1 |  | Festuca idahoensis Elmer | 0.5 | 0.3 |  |  |
| Geum macrophyllum Willd. |  |  | 0.1 | 0.1 |  | Festuca rubra L. | 0.3 | 0.3 |  |  |
| Glyceria striata (Lam.) A.S. Hitchc. | 3.6 | 0.9 | 0.6 | 0.3 |  | Fragaria virginiana Mill. ssp. platypetala (Rydb.) Staudt | 0.7 | 0.4 | 1.0 | 0.5 |
| Heracleum maximum W. Bartram | 0.1 | 0.1 |  |  |  | Galium boreale L. | 0.5 | 0.3 |  |  |
| Juncus ensifolius Wikstr. | 0.6 | 0.3 | 0.2 | 0.2 |  | Glyceria striata (Lam.) A.S. Hitchc. | 1.7 | 1.3 |  |  |
| Lolium multiflorum Lam. |  |  | 0.4 | 0.2 |  | Hypericum anagalloides Cham. & Schlecht. | 1.3 | 0.7 | 2.1 | 0.9 |
| Lolium perenne L. | 0.2 | 0.2 |  |  |  | Hypericum perforatum L. | 0.3 | 0.3 | 0.0 | 0.0 |
| Mentha canadensis L. | 4.0 | 0.8 | 0.1 | 0.1 |  | Juncus balticus Willd. | 5.8 | 2.1 | 6.0 | 1.7 |
| Mertensia ciliata (James ex Torr.) G. Don | 0.2 | 0.2 |  |  |  | Juncus ensifolius Wikstr. | 1.3 | 0.6 | 0.2 | 0.2 |
| Mimulus moschatus Dougl. ex Lindl. | 0.1 | 0.1 |  |  |  | Juncus nevadensis S. Wats. | 0.2 | 0.2 |  |  |
| Myosotis scorpioides L. | 32.1 | 4.1 | 8.0 | 1.4 |  | Lolium multiflorum Lam. | 0.3 | 0.3 |  |  |
| Phleum pratense L. | 6.6 | 1.6 | 17.1 | 2.4 |  | Mentha canadensis L. | 5.8 | 1.3 | 0.7 | 0.3 |
| Pinus contorta Dougl. ex Loud. | 5.6 | 3.3 |  |  |  | Medicago lupulina L. |  |  |  |  |
| Picea engelmannii Parry ex Engelm. |  |  | 1.6 | 1.6 |  | Erythranthe guttata (Fisch. ex DC.) G.L. Nesom | 0.2 | 0.2 | 0.1 | 0.1 |
| Plantago major L. |  |  | 2.3 | 0.9 |  | Mimulus moschatus Dougl. ex Lindl. | 0.1 | 0.1 |  |  |
| Populus trichocarpa Torr. & A. Gray | 0.6 | 0.6 |  |  |  | Phleum pratense L. | 0.1 | 0.1 |  |  |
| Potentilla gracilis Dougl. ex Hook. | 0.1 | 0.1 | 1.3 | 0.5 |  | Pinus contorta Dougl. ex Loud. | 6.3 | 3.2 |  |  |
| Poa pratensis L. | 8.9 | 2.4 | 36.5 | 2.8 |  | Potentilla gracilis Dougl. ex Hook. | 3.1 | 1.5 | 0.1 | 0.1 |
| Pseudotsuga menziesii (Mirbel) Franco |  |  | 1.3 | 1.3 |  | Polemonium occidentale Greene |  |  |  |  |
| Ribes aureum Pursh | 4.9 | 2.8 |  |  |  | Poa pratensis L. | 4.8 | 2.0 | 9.5 | 2.8 |
| Ribes lacustre (Pers.) Poir. | 1.7 | 1.0 |  |  |  | Prunella vulgaris L. |  |  | 2.9 | 1.3 |
| Rumex acetosella L. | 0.2 | 0.2 |  |  |  | Salix exigua Nutt. var. exigua | 2.5 | 1.6 |  |  |
| Micranthes odontoloma (Piper) A. Heller |  |  | 3.6 | 1.9 |  | Salix geyeriana Anderss. | 0.1 | 0.1 |  |  |
| Salix bebbiana Sarg. | 0.4 | 0.4 |  |  |  | Salix lasiandra Benth. var. caudata (Nutt.) Sudw. | 1.5 | 1.4 |  |  |
| Salix boothii Dorn | 0.1 | 0.1 |  |  |  | Solidago lepida DC.L. | 7.2 | 3.1 |  |  |
| Salix exigua Nutt. var. exigua | 0.2 | 0.2 |  |  |  | Eriocoma occidentalis (Thurb. ex S. Watson) Romasch. |  |  | 0.2 | 0.2 |
| Salix lasiandra Benth. var. caudata (Nutt.) Sudw. |  |  | 0.6 | 0.6 |  | Taraxacum officinale G.H. Weber ex Wiggers |  |  | 0.2 | 0.2 |
| Scirpus microcarpus J. Presl & C. Presl | 0.1 | 0.1 | 0.2 | 0.2 |  | Trifolium longipes Nutt. | 5.0 | 1.7 | 30.5 | 5.4 |
| Packera pseudaurea (Rydb.) W.A. Weber & Á. Löve | 0.7 | 0.4 |  |  |  |  |  |  |  |  |
| Senecio triangularis Hook. | 2.5 | 1.0 | 1.5 | 0.9 |  |  |  |  |  |  |
| Eriocoma occidentalis (Thurb. ex S. Watson) Romasch. | 0.3 | 0.2 |  |  |  |  |  |  |  |  |
| Symphoricarpos albus (L.) Blake | 2.9 | 2.0 |  |  |  |  |  |  |  |  |
| Taraxacum officinale G.H. Weber ex Wiggers | 0.3 | 0.2 |  |  |  |  |  |  |  |  |
| Trifolium repens L. | 5.2 | 2.5 | 19.4 | 3.1 |  |  |  |  |  |  |
| Veratrum californicum Dur. | 0.6 | 0.3 | 1.4 | 0.9 |  |  |  |  |  |  |

|  | Lower Swamp Creek | | | |  |  | Murderer's Creek | | | |
| --- | --- | --- | --- | --- | --- | --- | --- | --- | --- | --- |
|  | Exclosed | | Grazed | |  |  | Exclosed | | Grazed | |
|  | Mean | SE | Mean | SE |  |  | Mean | SE | Mean | SE |
| Achillea millefolium L. | 0.4 | 0.3 | 0.3 | 0.2 |  | Achillea millefolium L. | 0.3 | 0.3 | 1.5 | 1.1 |
| Agrostis stolonifera L. | 24.9 | 4.5 | 10.0 | 2.2 |  | Agrostis stolonifera L. | 1.5 | 0.5 | 1.3 | 0.5 |
| Agastache urticifolia (Benth.) Kuntze |  |  | 0.1 | 0.1 |  | Alnus incana (L.) Moench ssp. tenuifolia (Nutt.) Breitung | 8.1 | 3.4 |  |  |
| Alnus incana (L.) Moench ssp. tenuifolia (Nutt.) Breitung | 18.4 | 4.7 | 52.3 | 6.5 |  | Antennaria microphylla Rydb. |  |  | 0.2 | 0.2 |
| Amelanchier alnifolia (Nutt.) Nutt. ex M. Roemer |  |  | 0.3 | 0.3 |  | Symphyotrichum foliaceus Lindl. ex DC. | 9.2 | 3.2 | 2.0 | 1.2 |
| Carex geyeri Boott | 7.7 | 3.0 | 1.0 | 0.9 |  | Carex pellita Muhl. ex Willd.pellita | 2.4 | 1.5 |  |  |
| Carex microptera Mackenzie | 0.2 | 0.2 |  |  |  | Carex microptera Mackenzie | 0.5 | 0.3 |  |  |
| Carex nebrascensis Dewey | 1.3 | 1.3 |  |  |  | Carex nebrascensis Dewey | 5.2 | 1.8 | 26.5 | 5.7 |
| Carex utriculata Boott | 21.3 | 5.6 | 2.8 | 2.4 |  | Carex utriculata Boott | 20.5 | 4.4 | 4.6 | 2.3 |
| Carex stipata Muhl. ex Willd. | 0.8 | 0.5 |  |  |  | Cirsium remotifolium (Hook.) DC. var. odontolepis Petr. | 0.9 | 0.3 |  |  |
| Cirsium remotifolium (Hook.) DC. var. odontolepis Petr. | 2.4 | 0.7 |  |  |  | Dipsacus fullonum L. | 0.1 | 0.1 |  |  |
| Cerastium arvense L. | 0.8 | 0.4 |  |  |  | Epilobium ciliatum Raf. | 4.3 | 1.0 | 2.1 | 0.6 |
| Crataegus douglasii Lindl. |  |  | 2.1 | 1.3 |  | Equisetum arvense L. | 0.5 | 0.3 |  |  |
| Dipsacus fullonum L. | 0.3 | 0.3 |  |  |  | Erigeron philadelphicus L. | 0.4 | 0.3 | 0.1 | 0.1 |
| Epilobium ciliatum Raf. | 3.2 | 0.8 | 0.2 | 0.2 |  | Festuca rubra L. | 2.8 | 2.4 | 2.4 | 1.9 |
| Equisetum arvense L. | 1.9 | 0.9 |  |  |  | Galium aparine L. | 0.9 | 0.6 |  |  |
| Erigeron philadelphicus L. | 11.6 | 2.7 |  |  |  | Galium boreale L. | 0.2 | 0.2 |  |  |
| Fragaria virginiana Mill. ssp. platypetala (Rydb.) Staudt | 0.6 | 0.3 | 0.9 | 0.4 |  | Geum macrophyllum Willd. | 1.3 | 0.5 |  |  |
| Galium aparine L. | 3.1 | 1.5 | 0.5 | 0.3 |  | Glyceria striata (Lam.) A.S. Hitchc. | 13.5 | 3.5 | 2.3 | 1.1 |
| Galium boreale L. |  |  | 0.2 | 0.2 |  | Juncus balticus Willd. | 43.7 | 5.9 | 66.9 | 5.0 |
| Geum macrophyllum Willd. | 1.1 | 0.6 | 3.1 | 0.9 |  | Juncus nevadensis S. Wats. |  |  | 0.3 | 0.3 |
| Glyceria striata (Lam.) A.S. Hitchc. | 6.3 | 2.5 | 0.6 | 0.6 |  | Mentha canadensis L. | 6.6 | 2.3 |  |  |
| Juncus balticus Willd. | 14.3 | 4.4 | 0.3 | 0.3 |  | Medicago lupulina L. | 1.1 | 0.6 | 1.3 | 0.9 |
| Juncus ensifolius Wikstr. | 0.4 | 0.3 |  |  |  | Erythranthe guttata (Fisch. ex DC.) G.L. Nesom | 0.5 | 0.3 |  |  |
| Mentha canadensis L. | 0.8 | 0.4 | 0.2 | 0.2 |  | Myosotis scorpioides L. | 1.7 | 0.8 |  |  |
| Myosotis scorpioides L. |  |  | 1.1 | 0.4 |  | Plantago major L. | 0.5 | 0.4 |  |  |
| Phleum pratense L. | 1.8 | 0.6 | 7.2 | 1.4 |  | Potentilla gracilis Dougl. ex Hook. |  |  | 1.9 | 1.5 |
| Pinus contorta Dougl. ex Loud. | 1.2 | 0.9 | 2.9 | 2.1 |  | Polemonium occidentale Greene | 4.1 | 2.0 |  |  |
| Pinus ponderosa P.& C. Lawson |  |  | 0.3 | 0.2 |  | Poa pratensis L. | 22.6 | 4.3 | 24.3 | 3.6 |
| Plantago major L. |  |  | 0.5 | 0.3 |  | Nasturtium officinale W.T. Aiton | 1.3 | 0.6 |  |  |
| Potentilla gracilis Dougl. ex Hook. | 1.4 | 0.5 | 2.3 | 1.6 |  | Salix boothii Dorn | 2.3 | 1.1 | 0.1 | 0.1 |
| Poa pratensis L. | 6.2 | 2.2 | 48.5 | 3.7 |  | Salix geyeriana Anderss. | 0.2 | 0.2 |  |  |
| Ribes lacustre (Pers.) Poir. | 0.6 | 0.6 | 0.1 | 0.1 |  | Scirpus microcarpus J. Presl & C. Presl | 1.3 | 1.1 | 0.1 | 0.1 |
| Rosa woodsii Lindl. |  |  | 1.2 | 0.6 |  | Maianthemum stellatum (L.) Link |  |  | 0.9 | 0.6 |
| Salix geyeriana Anderss. | 3.0 | 1.7 |  |  |  | Trifolium longipes Nutt. |  |  | 0.6 | 0.6 |
| Scirpus microcarpus J. Presl & C. Presl | 2.8 | 1.9 | 0.6 | 0.6 |  | Veronica americana Schwein. ex Benth. | 2.6 | 0.8 | 2.0 | 0.8 |
| Packera pseudaurea (Rydb.) W.A. Weber & Á. Löve | 1.0 | 0.5 | 2.4 | 0.9 |  | Viola adunca Sm. | 0.7 | 0.4 | 0.2 | 0.2 |
| Maianthemum stellatum (L.) Link | 0.5 | 0.3 |  |  |  |  |  |  |  |  |
| Eriocoma occidentalis (Thurb. ex S. Watson) Romasch. |  |  | 1.5 | 0.9 |  |  |  |  |  |  |
| Symphoricarpos albus (L.) Blake | 0.4 | 0.4 | 2.1 | 1.0 |  |  |  |  |  |  |
| Taraxacum officinale G.H. Weber ex Wiggers |  |  | 0.1 | 0.1 |  |  |  |  |  |  |
| Thalictrum occidentale Gray | 0.3 | 0.2 | 0.5 | 0.3 |  |  |  |  |  |  |
| Trifolium repens L. |  |  | 1.5 | 0.7 |  |  |  |  |  |  |
| Urtica dioica L. | 0.3 | 0.3 |  |  |  |  |  |  |  |  |
| Veronica americana Schwein. ex Benth. | 0.1 | 0.1 |  |  |  |  |  |  |  |  |
| Veronica anagallis-aquatica L. | 1.7 | 0.7 | 0.7 | 0.4 |  |  |  |  |  |  |
| Veratrum californicum Dur. | 0.7 | 0.4 | 1.2 | 0.4 |  |  |  |  |  |  |

|  | Mid Fk John Day River | | | |
| --- | --- | --- | --- | --- |
|  | Exclosed | | Grazed | |
|  | Mean | SE | Mean | SE |
| Achillea millefolium L. | 10.5 | 3.2 | 0.7 | 0.4 |
| Agrostis stolonifera L. | 2.8 | 1.1 | 0.8 | 0.6 |
| Arnica chamissonis Less. | 2.9 | 1.0 | 1.5 | 0.6 |
| Symphyotrichum foliaceus Lindl. ex DC. | 0.3 | 0.2 |  |  |
| Carex pellita Muhl. ex Willd.pellita. | 30.2 | 4.6 | 10.4 | 3.4 |
| Carex nebrascensis Dewey | 7.9 | 2.6 |  |  |
| Carex utriculata Boott | 4.8 | 2.1 | 0.1 | 0.1 |
| Cerastium arvense L. |  |  | 1.6 | 0.6 |
| Cirsium remotifolium (Hook.) DC. var. odontolepis Petr. |  |  | 5.5 | 1.5 |
| Cornus sericea L. |  |  | 0.2 | 0.2 |
| Dactylis glomerata L. | 0.2 | 0.2 |  |  |
| Deschampsia cespitosa (L.) Beauv. | 15.1 | 3.4 |  |  |
| Eleocharis palustris (L.) Roemer & J.A. Schultes | 4.2 | 1.7 |  |  |
| Epilobium ciliatum Raf. | 3.3 | 0.7 | 0.6 | 0.5 |
| Equisetum arvense L. | 7.6 | 2.5 |  |  |
| Equisetum laevigatum A. Braun | 0.4 | 0.3 |  |  |
| Erigeron philadelphicus L. | 1.2 | 0.4 | 0.7 | 0.3 |
| Galium boreale L. | 0.3 | 0.3 | 0.2 | 0.2 |
| Geum macrophyllum Willd. | 0.2 | 0.2 |  |  |
| Glyceria striata (Lam.) A.S. Hitchc. | 1.7 | 0.5 |  |  |
| Hypericum anagalloides Cham. & Schlecht. | 0.2 | 0.2 |  |  |
| Iris missouriensis Nutt. | 0.5 | 0.4 |  |  |
| Juncus balticus Willd. | 13.4 | 3.9 | 1.5 | 0.9 |
| Lolium multiflorum Lam. | 1.3 | 1.0 | 0.4 | 0.4 |
| Mentha canadensis L. | 3.2 | 0.8 | 0.1 | 0.1 |
| Phleum pratense L. | 0.4 | 0.2 | 1.8 | 1.4 |
| Potentilla gracilis Dougl. ex Hook. | 5.8 | 2.2 | 0.1 | 0.1 |
| Poa pratensis L. | 15.2 | 3.8 | 64.2 | 3.2 |
| Ranunculus macounii Britt. | 0.4 | 0.3 |  |  |
| Salix geyeriana Anderss. |  |  | 0.1 | 0.1 |
| Salix lasiandra Benth. var. caudata (Nutt.) Sudw. | 0.1 | 0.1 | 0.3 | 0.3 |
| Saxifraga rhomboidea Greene | 0.4 | 0.4 |  |  |
| Sidalcea oregana (Nutt. ex Torr. & Gray) Gray | 0.1 | 0.1 |  |  |
| Maianthemum stellatum (L.) Link | 1.3 | 0.9 | 0.1 | 0.1 |
| Solidago lepida DC. | 15.4 | 4.4 | 11.3 | 2.6 |
| Taraxacum officinale G.H. Weber ex Wiggers | 0.3 | 0.2 | 3.4 | 0.6 |
| Verbascum thapsus L. |  |  | 0.9 | 0.4 |
